# Emerging Viral Zoonoses: Epidemiology, Vaccination Strategies, and Implications for Global Public Health

**DOI:** 10.3390/vaccines14070560

**Published:** 2026-06-25

**Authors:** Julia Dulska, Marek Fol, Magdalena Druszczynska

**Affiliations:** Department of Immunology and Infectious Biology, Faculty of Biology and Environmental Protection, University of Lodz, Banacha 12/16, 90-237 Lodz, Poland; julia.dulska@edu.uni.lodz.pl (J.D.); marek.fol@biol.uni.lodz.pl (M.F.)

**Keywords:** zoonotic viruses, emerging infectious diseases, vaccination strategies, One Health

## Abstract

**Background/Objectives**: Emerging viral zoonoses represent a growing threat to global public health, with most newly emerging infectious diseases originating from animal reservoirs. Recent outbreaks of monkeypox, Ebola virus disease, Marburg virus disease, Rift Valley fever, and avian influenza highlight the capacity of zoonotic viruses to cross species barriers, spread internationally, and generate substantial health, social, and economic consequences. This review examines the ecological, epidemiological, and biological determinants of viral zoonotic emergence and transmission, with particular emphasis on vaccination and outbreak prevention strategies. **Methods**: A structured narrative review was conducted using a predefined literature search strategy across major scientific databases. Peer-reviewed epidemiological, clinical, and public health publications published between January 2000 and February 2026 were screened and selected according to predefined relevance criteria. **Results**: The emergence of viral zoonoses is driven by complex interactions among animal reservoirs, environmental and climatic changes, human behavior, and viral adaptation. Although transmission pathways and clinical outcomes differ among pathogens, common determinants of spillover and outbreak amplification were identified. Current evidence supports the importance of integrated surveillance, genomic monitoring, vaccination strategies, and community engagement as key components of preparedness and response. Emerging preventive approaches targeting pathogen transmission, including transmission-blocking strategies and vector-associated microbiota interventions, may provide additional opportunities for disease control. **Conclusions**: Strengthening preparedness for emerging viral zoonoses requires coordinated One Health approaches integrating human, animal, and environmental health. Future priorities include the development of next-generation vaccines, expansion of digital and genomic surveillance systems, improved equitable access to vaccines, and innovative interventions aimed at reducing zoonotic spillover and interrupting pathogen transmission.

## 1. Introduction

Humans are continuously exposed to a wide range of infectious agents originating from the environment, animals, and other humans. Emerging and re-emerging pathogens rapidly adapt to changing ecological conditions, developing mechanisms that enable host switching, persistence, and efficient transmission. Among these, zoonotic pathogens—defined as microorganisms transmitted between vertebrate animals and humans—represent a major and growing global public health concern [1,2]. It is estimated that approximately 60–75% of known human pathogens and nearly 75% of emerging infectious diseases (EIDs) originate from animal reservoirs, highlighting the central role of zoonotic transmission in the development of epidemics and pandemics [2,3,4]. Viral pathogens are particularly prone to cross-species transmission due to high mutation rates, genetic recombination, and rapid adaptation to new hosts and environmental pressures [5].

Historically, zoonotic diseases have profoundly shaped human societies, affecting population health, economies, and demographic structures. Classical examples include tuberculosis, plague, avian influenza, and rabies [6]. Tuberculosis caused by members of the *Mycobacterium tuberculosis* complex may originate from both human- and animal-adapted strains, demonstrating the long-standing interconnection between human and animal health [7,8]. Plague, caused by *Yersinia pestis*, was responsible for multiple pandemics, including the Black Death, illustrating the devastating consequences of zoonotic transmission [9,10]. Avian influenza viruses, particularly highly pathogenic strains such as H5N1, continue to pose pandemic risks due to their ability to reassort genetically and infect humans following exposure to infected poultry [11,12,13]. Rabies remains one of the most lethal viral zoonoses, with an almost 100% fatality rate once clinical symptoms appear [14,15,16].

The frequency of zoonotic spillover events has increased in recent decades, largely due to anthropogenic environmental changes such as deforestation, biodiversity loss, climate change, agricultural intensification, wildlife trade, and urban expansion [17,18,19]. These processes increase contact between humans and potential animal reservoirs, facilitating viral adaptation and interspecies transmission. Wildlife species, particularly bats, rodents, and birds, serve as important reservoirs for emerging viral pathogens, while domestic animals may act as intermediate or amplifying hosts enabling viral evolution and transmission to humans [20,21]. Globalization, urbanization, and increased international mobility further accelerate pathogen dissemination, increasing the probability that local outbreaks evolve into regional epidemics or global pandemics [22].

Recent public health emergencies, including the COVID-19 pandemic, Ebola outbreaks, and the 2022 mpox epidemic, highlight the continuing threat posed by emerging viral zoonoses [23,24,25]. These events demonstrate how rapidly zoonotic viruses can spread in highly interconnected societies and emphasize the importance of effective prevention strategies. Vaccination remains one of the most effective tools for controlling infectious disease outbreaks, significantly reducing morbidity and mortality [26]. Advances in vaccine technologies, including mRNA and viral vector platforms, have demonstrated considerable potential for rapid response to emerging pathogens [27,28]. However, major challenges persist, including unequal global vaccine distribution, limited availability of vaccines for certain zoonotic pathogens, and insufficient early-warning surveillance systems capable of detecting spillover events [29].

This review provides a comprehensive overview of major emerging viral zoonoses, including monkeypox (mpox), Ebola virus disease, Rift Valley fever, Marburg virus disease, and avian influenza, focusing on their epidemiology, transmission dynamics, and implications for global public health. Particular attention is given to ecological, epidemiological, and biological determinants of zoonotic emergence, as well as current vaccination strategies and public health interventions aimed at outbreak prevention and preparedness. Understanding the shared mechanisms underlying zoonotic spillover and epidemic spread is essential for improving surveillance systems, guiding vaccine development, and strengthening global preparedness for future emerging infectious disease threats. Although the reviewed zoonoses differ in their reservoirs, transmission pathways, and clinical manifestations, they share several common mechanisms that drive zoonotic emergence and outbreak propagation. Spillover events typically occur at the human–animal–environment interface and are facilitated by ecological disruption, land-use change, deforestation, agricultural intensification, wildlife trade, and increasing human encroachment into natural habitats. These processes increase opportunities for contact between humans, domestic animals, and wildlife reservoirs, thereby enhancing the likelihood of cross-species transmission. Furthermore, despite differences in viral taxonomy and transmission routes, these pathogens are linked by common ecological, environmental, and socio-economic pressures that facilitate both spillover and epidemic spread. Following spillover, pathogen emergence is influenced by factors such as viral genetic adaptation, host susceptibility, population density, human mobility, and healthcare system capacity. Outbreak dynamics are further shaped by delays in detection, limited surveillance infrastructure, inadequate risk communication, and insufficient access to preventive measures, including vaccination. Collectively, these shared drivers highlight the interconnected ecological, epidemiological, biological, and social determinants of zoonotic disease emergence and reinforce the need for integrated One Health approaches to prevention, preparedness, and response. These common pathways suggest that effective prevention strategies should focus not only on pathogen-specific interventions but also on addressing the broader environmental and societal factors that contribute to the emergence and spread of zoonotic diseases.

## 2. Materials and Methods

This structured narrative review was conducted using a predefined literature search strategy to identify studies addressing the epidemiology of emerging viral zoonoses, transmission dynamics, determinants of zoonotic spillover, and vaccination strategies for outbreak prevention and control, with particular focus on mpox, Ebola virus disease, Marburg virus disease, Rift Valley fever, and avian influenza. Electronic databases, including PubMed, PubMed Central, Scopus, Web of Science, ScienceDirect, and Google Scholar, were searched for publications published from January 2000 to 12 February 2026. The final literature search was conducted on 15 February 2026. The search strategy incorporated keywords related to zoonotic viral infections, epidemiology, transmission, and immunization, including “zoonotic viruses”, “spillover”, “transmission dynamics”, “monkeypox”, “Ebola virus”, “Marburg virus”, “Rift Valley fever virus”, “avian influenza”, “vaccination strategies”, and “vaccine effectiveness”. Boolean operators (AND, OR) were applied where appropriate to combine and refine search terms. Publications were eligible for inclusion if they were peer-reviewed original research articles, systematic reviews, narrative reviews, epidemiological investigations, clinical studies, surveillance reports, or reports issued by recognized international public health organizations. Only English-language publications were included. Conference abstracts without full-text availability, letters to the editor, duplicate records, non-peer-reviewed publications, and studies not directly relevant to the objectives of this review were excluded. Retrieved records were assessed on the basis of title and abstract screening, followed by full-text evaluation where necessary. Studies were selected according to their relevance to zoonotic emergence, transmission pathways, outbreak dynamics, prevention strategies, vaccination approaches, and public health preparedness. Given the broad scope of the topic and the heterogeneity of pathogens, study designs, and outcomes, a formal systematic review methodology and quantitative synthesis were not performed. As this study was designed as a structured narrative review rather than a systematic review, a formal PRISMA-guided screening workflow was not applied. Instead, findings were synthesized qualitatively to provide an integrated overview of current evidence, identify common drivers of zoonotic emergence, and highlight key challenges and future directions for prevention and control.

## 3. Drivers of Viral Zoonotic Emergence

**Figure 1:**
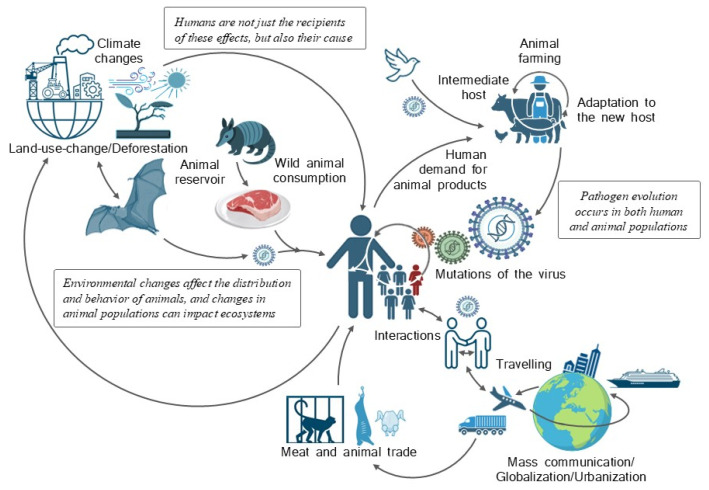
Conceptual framework illustrates the bidirectional interactions and feedback loops among environmental, animal, and human components driving zoonotic pathogen emergence. Environmental change, animal reservoirs, and human activities interact dynamically through the human–animal–environment interface, facilitating pathogen spillover, adaptation, transmission, and subsequent feedback effects on ecosystems and host populations. Environmental changes affect the distribution and behavior of animals, and changes in animal populations can impact ecosystems based on [30,31,32]. The figure was created in part using BioRender.com.

The emergence of viral zoonoses is driven by complex interactions among ecological, biological, and socio-economic factors (Figure 1). Understanding these drivers is critical for predicting spillover events and designing effective public health interventions.

### 3.1. Animal Reservoirs and Host–Pathogen Interactions

Wildlife species such as bats, rodents, and birds are recognized as key reservoirs for many emerging viruses due to their high population densities, species diversity, and ability to harbor multiple pathogens asymptomatically [20,21,33]. Bats are reservoirs for filoviruses such as Ebola and Marburg, as well as henipaviruses; rodents harbor hantaviruses and arenaviruses; and birds carry avian influenza viruses [34,35,36]. Viral adaptation to new hosts involves genetic changes—including point mutations, recombination, and reassortment—that increase infectivity, host range, and transmission potential [5,37]. These host–pathogen interactions determine the likelihood of spillover into human populations and the risk of epidemic or pandemic spread.

### 3.2. Environmental and Ecological Determinants

Environmental and ecological changes strongly influence the risk of zoonotic emergence. Climate change, including rising temperatures and altered precipitation patterns, shifts the geographic range, abundance, and seasonal activity of animal reservoirs and arthropod vectors, thereby increasing opportunities for human exposure [38,39]. Habitat fragmentation and biodiversity loss reduce ecosystem resilience, alter predator–prey relationships, and elevate contact between humans and wildlife, facilitating pathogen spillover across species barriers [19,40]. Agricultural intensification, including high-density livestock farming and encroachment into natural habitats, further amplifies interfaces between humans, domestic animals, and wildlife, creating conditions conducive to pathogen emergence [41,42].

In addition to environmental and ecological drivers, pathogen transmission is also influenced by biological factors intrinsic to vectors. Vector competence is determined not only by external ecological conditions but also by the vector’s immune responses, genetic background, and interactions with associated microbial communities. Increasing evidence indicates that vector competence such as the ability of a vector to acquire, maintain, and transmit a pathogen is shaped not only by external environmental conditions but also by the vector’s immune responses, genetic background, and the composition of its associated microbiota. Experimental studies have demonstrated that perturbation of microbial communities in arthropod vectors may reduce pathogen colonization and transmission, highlighting microbiota as a potential target for future transmission-blocking interventions [43,44]. Interactions between environmental changes and vector biology may further influence the geographic distribution and transmission capacity of vector populations. The interplay between environmental pressures and vector biology underscores the multifactorial nature of zoonotic disease emergence and highlights emerging opportunities for integrated vector-targeted control strategies within the One Health framework [43,44].

### 3.3. Human Behavior and Socio-Economic Factors

Human behavior and socio-economic activities are major determinants of zoonotic risk. International travel and trade accelerate pathogen spread, potentially transporting infections across continents within hours [45]. Urbanization increases population density and alters local ecosystems, creating novel human–animal interactions [46]. Direct contact with animals through livestock handling, hunting, wildlife trade, or consumption of bushmeat facilitates cross-species transmission [47,48]. Poverty, limited healthcare access, and inadequate biosecurity measures exacerbate exposure and hinder outbreak response.

### 3.4. Mechanisms of Spillover and Human-to-Human Transmission

Zoonotic spillover occurs when pathogens overcome interspecies barriers. Viruses may acquire preadaptive mutations in their natural reservoirs that allow initial infection in humans, followed by adaptive mutations that facilitate replication and human-to-human transmission [5,49,50]. Ecological disruption, increased human–wildlife contact, and high population density further enhance spillover probability [22].

### 3.5. Pathways of Pathogen Transmission

Microorganisms can be transmitted in many ways, ranging from indirect involvement of biological vectors spreading infection between hosts, to direct contact with an infected animal, or through contaminated food and water [51].

Direct contact transmission is a major pathway for zoonotic pathogens, occurring when humans interact with infected animals or their secretions, including saliva, urine, feces, blood, milk, nasal discharge, amniotic fluid, or open wounds [50,52,53]. Pathogens must first overcome interspecies barriers to infect humans, often acquiring preadaptive mutations in their natural reservoirs that enable spillover, followed by adaptive mutations that allow survival and replication in the new host [50,54]. Rabies provides a clear example of this mechanism. The virus, belonging to the *Rhabdoviridae* family, is transmitted through bites from infected bats, dogs, monkeys, wolves, foxes, squirrels, or other mammals. Viral particles in saliva enter the human bloodstream and invade the central nervous system, causing acute inflammation of the brain and spinal cord and almost invariably resulting in death if untreated [2]. Transmission can also occur via broken skin or mucous membranes contacting contaminated secretions. Direct contact with animals or their biological fluids plays a critical role in the emergence of zoonotic epidemics and pandemics, highlighting the importance of limiting human–animal interactions, implementing safe handling practices, and enhancing surveillance in both wildlife and domestic animal populations to reduce infection risk.

Indirect transmission occurs when pathogens are spread without direct contact with an infected host, primarily via biological vectors or contaminated environments. Biological vectors—most commonly blood-feeding invertebrates such as mosquitoes, ticks, fleas, and bedbugs—can transmit pathogens externally, on contaminated mouthparts, or internally after replication within the vector [55,56,57]. Long-term evolutionary associations between pathogens and vectors may result in transstadial transmission (from immature to adult stages) or transovarial transmission (from female vectors to offspring), while horizontal transmission among vector populations can occur through sexual contact or simultaneous feeding on vertebrate hosts [58]. Vector-borne transmission plays a critical role in the spread of many viral zoonoses, including Rift Valley fever virus and various arboviruses.

Recent evidence suggests that vector competence is influenced not only by pathogen–vector interactions but also by biological factors intrinsic to the vector, including immune responses and the composition of vector-associated microbiota. Microbial communities inhabiting mosquitoes, ticks, and other arthropod vectors can affect pathogen acquisition, colonization, replication, and transmission efficiency, thereby influencing disease dynamics and outbreak potential [43]. Consequently, microbiota-based interventions and transmission-blocking approaches are increasingly being explored as complementary strategies for reducing pathogen transmission and improving the control of vector-borne diseases [43,44]. Although these approaches remain largely experimental, they represent promising future directions for integrated zoonotic disease prevention within the One Health framework.

Environmental contamination represents another important indirect transmission pathway. Accumulated feces, urine, or other bodily fluids containing pathogens can contaminate water, soil, or air, which then act as reservoirs for infectious agents [59]. Humans interacting with contaminated environments may become infected, as seen in outbreaks of waterborne diseases such as cholera (*Vibrio cholerae*), tularemia (*Francisella tularensis*), or airborne infections such as Q fever (*Coxiella burnetii*) and avian influenza viruses. Indirect transmission thus encompasses both vector-mediated spread and environmental reservoirs, highlighting the diverse mechanisms by which zoonotic pathogens reach human populations [60,61,62,63,64].

Indirect environmental and foodborne transmission represents a major pathway for zoonotic pathogens, complementing vector-mediated spread. Foodborne transmission is a significant route, with meat, dairy, fruits, and vegetables serving as vehicles for pathogens. Increased meat consumption, population growth, and globalization can sustain chronic sources of contamination [65]. Several zoonotic bacteria are monitored annually in public health programs, including *Campylobacter*, *Salmonella*, *Listeria*, Shiga toxin–producing *E. coli* (STEC), and bovine tuberculosis (*Mycobacterium bovis*, *M. caprae*) [66].

Contaminated water is another important source of infection. Water reservoirs can harbor microorganisms such as *Vibrio cholerae*, the causative agent of cholera, which thrives under poor sanitary conditions and causes severe gastrointestinal disease [59]. Water contaminated with carcasses or feces from infected animals can also transmit *Francisella tularensis*, the agent of tularemia, which is highly resistant to environmental stressors and associated with high mortality, leading to its classification as a potential biological weapon [57]. Airborne transmission represents an additional critical route for zoonotic pathogens. Droplet-mediated spread facilitates infection by agents including avian influenza viruses, *Coxiella burnetii* (Q fever), SARS-CoV-2, and MERS-CoV [2,59,64].

### 3.6. Risk Factors for Transmission

Environmental, biological, and socio-economic drivers of pathogen transmission are multifactorial and closely interrelated. Direct contact with wildlife, including hunting and the trade of animals in markets—often under unhygienic conditions—significantly increases human exposure to zoonotic pathogens [67,68]. Domestic animals can act as “bridges,” facilitating cross-species transmission that might otherwise be evolutionarily constrained [68].

Climate change and urbanization further modify habitats and wildlife movement, increasing interactions between humans and novel pathogens. Global warming alters geographical ranges of wildlife and reduces natural habitats, forcing animals to migrate in search of food and shelter, which exposes them to new pathogens and facilitates their spread [69]. Socio-economic factors, including population density, globalization, and increased human mobility, accelerate pathogen dissemination. Local spillover events can rapidly develop into regional or global threats due to international travel and trade, highlighting the intersection between ecological disruption and human behavior in driving zoonotic emergence [31,70]. All these environmental, biological, and socio-economic factors underscore the complex, multifactorial nature of viral zoonotic emergence. Effective mitigation requires integrated strategies combining surveillance, ecological management, wildlife monitoring, and targeted public health interventions to reduce the risk of spillover and subsequent outbreaks.

## 4. Selected Emerging Viral Zoonoses

### 4.1. Monkeypox

#### 4.1.1. Epidemiology

Monkeypox virus (MPXV) was first identified in 1958 in captive crab-eating macaques (*Macaca fascicularis*) in Denmark [71,72,73,74,75,76,77]. The first confirmed human case was reported in 1970 in the Democratic Republic of Congo (DRC), marking the beginning of recognized human mpox infections. Since then, MPXV has remained endemic in Central and West Africa, with the majority of cases reported in the DRC and surrounding regions, where zoonotic spillover from wildlife reservoirs is considered the primary source of infection [77,78,79]. Over time, sporadic outbreaks and increasing geographic spread have been documented, culminating in the 2022 multicountry outbreak, which marked the first large-scale global dissemination outside endemic regions not directly linked to travel or imported animals.

**Figure 2:**
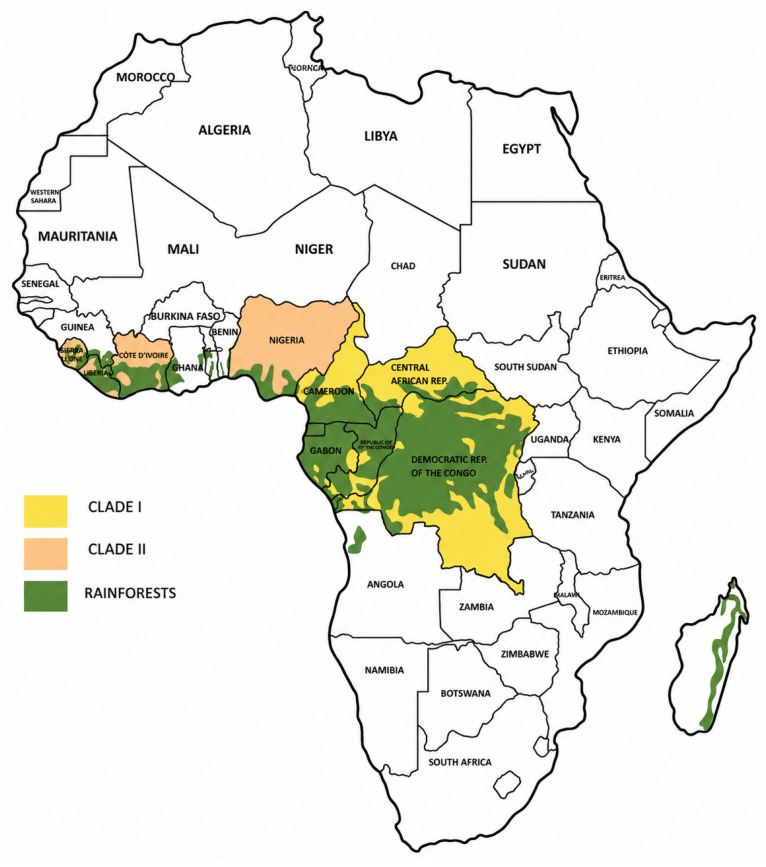
Geographic distribution of MPXV clades in Africa. Clade I (Congo Basin) predominates in Central Africa and is associated with more severe disease, whereas Clade II (West African clade) occurs mainly in West Africa, with subclade IIb responsible for the global outbreaks reported since 2022. based on [77,78,79,80,81,82,83].

Two genetically and epidemiologically distinct clades are recognized: clade I (Central African/Congo Basin) and clade II (West African), with further subdivision into subclades Ia, Ib, IIa, and IIb (Figure 2). Clade I is associated with higher virulence and mortality, whereas clade IIb has been responsible for the global outbreak since 2022 and is characterized by enhanced human-to-human transmission. The outbreak prompted the World Health Organization to declare a public health emergency of international concern on 14 August 2024 [80].

#### 4.1.2. Transmission

**Figure 3:**
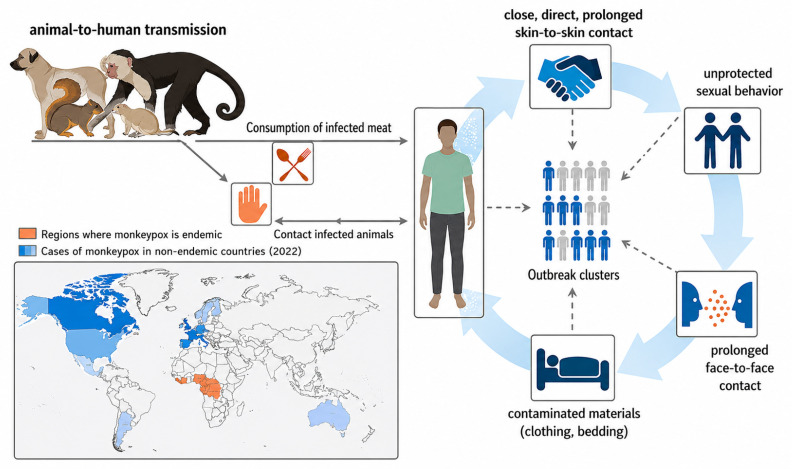
The transmission cycle of monkeypox. The virus circulates in reservoir animals, particularly rodents and non-human primates, and can be transmitted to humans through direct contact with infected animals or their bodily fluids. Human-to-human transmission occurs through contact with skin lesions, respiratory droplets during prolonged close contact, and contaminated materials such as clothing or bedding (fomites). The figure also indicates endemic regions in Central and West Africa and illustrates the global spread observed after the 2022 multicountry outbreak based on [88,89,90,91]. The figure was created in part using BioRender.com.

MPXV transmission is multifactorial, encompassing both animal-to-human (zoonotic) and human-to-human pathways (Figure 3). Traditionally considered a zoonosis, MPXV is transmitted through direct contact with infected animals such as rodents or monkeys, including bites, scratches, and the handling of infected meat [84,85]. Human-to-human transmission occurs via droplets, contact with body fluids, skin lesions, and contaminated fomites such as bedding or clothing. Sexual transmission, particularly among men who have sex with men (MSM), has emerged as a significant route during recent outbreaks [86]. Vertical transmission from mother to fetus has been documented in rare cases, with severe outcomes including miscarriage or congenital mpox syndrome [87]. The virus is capable of surviving on surfaces for extended periods, reflecting its stability in the environment. Transmission risk is further influenced by socio-behavioral factors, population density, and immunological susceptibility.

#### 4.1.3. Clinical Features

The incubation period for MPXV typically ranges from 7 to 14 days, though it can extend from 4 to 21 days [92]. Infection progresses in two phases. The prodromal phase lasts 1–5 days and includes fever, headache, myalgia, malaise, and lymphadenopathy, which distinguishes mpox from similar viral infections such as smallpox or chickenpox (Table 1). The rash phase lasts 2–4 weeks and is characterized by sequential skin lesions—macules, papules, vesicles, and pustules—that may be painful or pruritic, eventually forming scabs that leave hypo- or hyperpigmented scars [84]. Severe or atypical manifestations may involve internal organs or bones, particularly in immunocompromised individuals or pregnant women. Male predominance has been consistently reported in mpox outbreaks. Although epidemiological factors appear to play a major role, sex-based differences in immune responses have been proposed as a potential contributing factor, with androgens generally exerting immunosuppressive effects that may influence host susceptibility to viral infections [93,94,95].

#### 4.1.4. Vaccination and Treatment

Immunity to MPXV involves both innate and adaptive immune responses [92,100]. The virus employs multiple mechanisms to evade host defenses, including inhibition of apoptosis and interference with antigen presentation [100]. Historically, smallpox vaccination conferred cross-protection against MPXV. First- and second-generation live vaccines effectively reduced disease severity but had significant side effects. Third-generation vaccines, such as Modified Vaccinia Ankara–Bavarian Nordic (MVA-BN), are non-replicating and safer, offering protection to high-risk populations, including immunocompromised individuals and pregnant women [92,101]. MVA-BN is administered in two doses, generating immunity against orthopoxviruses, and is marketed as Imvanex (EU), Jynneos (USA), and Imvamune (Canada) [99,102,103,104,105,106]. The LC16m8 vaccine (also known as LC16 or LC16 KMB) developed in Japan represents an alternative option but is less widely used and not EMA-approved (Table 2). Vaccination remains a cornerstone of mpox prevention, particularly in outbreak settings, alongside infection control measures such as hygiene, surface disinfection, isolation of cases, and public education. Antiviral therapies, including tecovirimat (TPOXX^®^), brincidofovir, and cidofovir, are reserved for severe or complicated cases, particularly in vulnerable populations [107].

#### 4.1.5. Future Challenges

Despite the demonstrated effectiveness of available vaccines, several challenges remain in mpox prevention strategies. These include uncertainties regarding the duration of vaccine-induced immunity, limited access to vaccines in endemic regions, and logistical and infrastructural barriers to timely vaccine deployment during outbreaks. Addressing these gaps is essential for optimizing vaccination strategies and improving outbreak preparedness and response in both endemic and non-endemic settings.

### 4.2. Avian Influenza

#### 4.2.1. Epidemiology

Avian influenza viruses (AIVs) are members of the genus Influenza A in the family Orthomyxoviridae and possess a segmented negative-sense RNA genome, which enables frequent genetic reassortment and contributes to their pandemic potential [108]. The natural reservoir of AIVs is wild aquatic birds, particularly waterfowl, which support long-term viral circulation without causing significant disease. Domestic poultry species act as intermediate amplifying hosts and play a central role in zoonotic transmission to humans. Human avian influenza infections have been documented sporadically since the late 20th century and are distributed globally, with the highest burden historically reported in Asia, although increasing numbers of cases have been identified in Europe, Africa, and other regions due to intensified poultry trade and migratory bird pathways. These infections are typically associated with direct or indirect exposure to infected birds or contaminated poultry environments.

Among the numerous AIV subtypes, H5N1, H7N9, and H9N2 are of greatest public health concern due to their confirmed zoonotic potential and repeated emergence in human populations (Table 3). H5N1 is associated with highly severe disease and high case fatality, H7N9 emerged in China in 2013 and caused multiple epidemic waves in humans, and H9N2 is widely endemic in poultry populations and serves as an important genetic donor in reassortment events contributing to the emergence of novel zoonotic strains [109,110,111]. Collectively, these features highlight the ongoing evolutionary risk posed by AIVs and their potential to generate viruses with pandemic capacity. A notable recent development in the epidemiology of avian influenza has been the emergence of highly pathogenic avian influenza A (H5N1) infections in dairy cattle. Since 2024, outbreaks reported in the United States have demonstrated that H5N1 viruses can infect and circulate among bovine populations, considerably expanding the recognized host range of the virus. Infected cattle typically presented with reduced milk production, decreased feed intake, and mild systemic symptoms, while viral RNA and infectious virus were detected in milk and respiratory secretions. Although human infections associated with exposure to infected cattle have remained sporadic, these events have raised concerns regarding viral adaptation to mammalian hosts and increased opportunities for cross-species transmission. Furthermore, recent detections of H5N1 in various mammalian species in Europe and other regions highlight the growing importance of integrated One Health surveillance and continued monitoring of viral evolution at the animal–human interface [112,113,114].

#### 4.2.2. Transmission

Human infection with AIVs typically occurs through direct or indirect exposure to infected birds, their secretions, or contaminated environments such as live bird markets and poultry farms. Transmission remains predominantly zoonotic, and sustained human-to-human transmission has not been established for most subtypes. However, limited familial clusters of H7N9 infection suggest partial adaptation and ongoing evolutionary risk [122,123,124,125].

The segmented genome of influenza A viruses enables reassortment during co-infection of host cells, resulting in novel genotypes with altered host range, transmissibility, and virulence. Such reassortment events, particularly involving H9N2 internal genes, have been implicated in the emergence of human-infecting strains such as H7N9.

#### 4.2.3. Clinical Features

Human infection with AIVs ranges from mild conjunctivitis or influenza-like symptoms to severe pneumonia, acute respiratory distress, and multiorgan involvement [110]. Host immune responses involve both innate mechanisms, such as interferon signalling and NK cell activity, and adaptive responses including HA-specific antibodies and CD8+ T-cell mediated immunity, which are critical for viral clearance and recovery [126,127]. The risk of severe disease increases with age, co-morbidities, and lack of pre-existing immunity.

#### 4.2.4. Vaccination and Prevention

Vaccination remains the cornerstone of influenza prevention and pandemic preparedness. Seasonal influenza vaccines are standard for seasonal epidemiology, but they do not reliably protect against emerging avian subtypes [114]. Specific vaccines targeting AIVs have been developed and evaluated in both preclinical and clinical settings (Table 4). For example, clinical studies have assessed the immunogenicity and safety of H5N1 vaccine candidates in humans, reporting variable antibody responses to inactivated formulations, which underscores the need for optimized vaccine designs for effective immunoprotection in diverse populations [128,129]. Although no widely deployed human AIV vaccine exists, research on novel platforms, including mRNA-based vaccines encoding HA antigens that have shown protective efficacy in animal models against divergent strains—offers promise for future human vaccines capable of eliciting robust humoral and cellular responses across antigenically distinct AIV variants [130,131]. Continued monitoring of viral evolution, coupled with vaccine research and immunological studies, is essential to reduce the risk of a future avian influenza pandemic. Global efforts to advance vaccine development include WHO-led initiatives to accelerate mRNA vaccine platforms specific to H5N1 and other AIV subtypes, enhancing pandemic preparedness by enabling scalable production and broader access, especially in low- and middle-income countries [131]. In addition, WHO’s Global Influenza Surveillance and Response System (GISRS) supports the selection and distribution of candidate vaccine viruses for outbreak response, particularly for H7N9, and provides technical guidance for vaccine development in anticipation of zoonotic threats [132].

#### 4.2.5. Future Challenges

Despite significant progress in influenza vaccine research, the development of a truly universal influenza vaccine remains challenging. Major obstacles include the high antigenic variability of influenza viruses, driven by continuous antigenic drift and occasional antigenic shift, as well as immune imprinting that may limit the breadth of vaccine-induced protection. In addition, the durability of cross-protective immune responses remains insufficient for long-term protection against divergent strains. These evolutionary dynamics underscore the need for continuously updated vaccine strategies and highlight the complexity of achieving long-term, broad-spectrum immunization against influenza viruses.

### 4.3. Hemorrhagic Fevers—Ebola and Marburg Virus Diseases

#### 4.3.1. Epidemiology

Ebola virus disease (EVD) and Marburg virus disease (MVD) are severe viral hemorrhagic fevers caused by distinct members of the family *Filoviridae*. EVD is caused by viruses of the genus Ebolavirus, with Zaire ebolavirus (EBOV) responsible for the largest and most severe outbreaks, while MVD is caused by *Marburgvirus*, primarily Marburg virus (MARV) [144,145,146].

Both diseases are zoonotic in origin and are primarily associated with African ecosystems. Their natural reservoirs are linked to fruit bats. Although the definitive reservoir for Ebola viruses has not been fully confirmed, ecological and serological evidence strongly implicates bat species within the family *Pteropodidae*. In contrast, *Rousettus aegyptiacus* (Egyptian fruit bat) has been identified as the confirmed reservoir host for MARV, with human infections frequently associated with exposure to bat-inhabited caves and mines [147,148,149]. Sporadic spillover events from wildlife into humans represent the primary mechanism of emergence, followed by limited human-to-human transmission chains that can result in large outbreaks under conditions of weak healthcare infrastructure.

#### 4.3.2. Transmission

Transmission to humans typically occurs through direct contact with infected animals or their secretions, including blood, tissues, or body fluids. Human-to-human transmission then proceeds primarily via direct contact with blood or bodily fluids such as saliva, urine, sweat, vomit, breast milk, and semen or through contaminated objects, bedding, and surfaces. Both filoviruses are not transmitted through casual airborne spread, although rare aerosol exposure under experimental conditions has been described [144,146,150,151,152,153,154,155,156,157].

#### 4.3.3. Clinical Features

Clinically, both diseases begin with nonspecific symptoms, including high fever, severe headache, muscle pain, and malaise, often progressing rapidly to gastrointestinal symptoms and systemic involvement (Table 5). Severe cases develop hemorrhagic manifestations, organ dysfunction, coagulation disorders, and hemodynamic collapse, significantly increasing fatality risk. EVD outbreaks have exhibited case fatality rates ranging from about 25% to 90%, depending on the viral species, outbreak setting, and access to medical care; MARV outbreaks similarly display high mortality, with reported rates up to 88% in settings lacking advanced supportive care [158,159,160].

#### 4.3.4. Vaccination and Treatment

Management of both diseases remains largely supportive, focusing on fluid resuscitation, hemodynamic support, oxygenation, and treatment of secondary infections. For EVD, prophylactic vaccination has been a major advancement: the recombinant vesicular stomatitis virus-Zaire ebolavirus vaccine (Ervebo) and the two-component Zabdeno/Mvabea regimen are approved and recommended as part of outbreak response and for at-risk health workers [161,162]. In contrast, no licensed vaccine or broadly effective prophylaxis exists for MVD at present, although investigational vaccines and therapeutics are under development and evaluation [163,164].

#### 4.3.5. Future Challenges

Despite progress in Ebola prevention and control, significant challenges remain. These include maintaining vaccination coverage in endemic regions, ensuring rapid deployment during outbreaks, and strengthening surveillance systems for early detection of spillover events. For Marburg virus disease, the absence of an approved vaccine and limited therapeutic options represent major gaps in preparedness. More broadly, the unpredictable nature of filovirus emergence from wildlife reservoirs highlights the need for strengthened One Health surveillance systems, improved ecological monitoring of bat populations, and enhanced global coordination for outbreak response.

### 4.4. Rift Valley Fever

#### 4.4.1. Epidemiology

Rift Valley Fever (RVF) is caused by a virus of the genus *Phlebovirus* in the family *Phenuiviridae*. It is an enveloped virus with a segmented single-stranded negative-sense RNA genome, which allows genetic reassortment under co-infection conditions, although reassortment events in RVFV are less frequent than in influenza viruses [165]. The genome encodes structural proteins, including the nucleoprotein (N), glycoproteins Gn and Gc responsible for host cell entry, and non-structural proteins involved in immune evasion and modulation of host antiviral responses [166,167]. RVFV is primarily maintained in nature via a mosquito–vertebrate cycle, with wild and domestic ruminants serving as amplifying hosts (Table 6). RVF is endemic in sub-Saharan Africa, with outbreaks commonly associated with periods of heavy rainfall that favor mosquito breeding. Until 2000, RVF outbreaks were largely restricted to Africa, but cases have since been reported in the Arabian Peninsula, including Saudi Arabia and Yemen [168]. High-risk groups include farmers, herders, veterinarians, and slaughterhouse workers.

#### 4.4.2. Transmission

RVFV transmission occurs primarily through mosquito vectors belonging to the genera *Aedes*, *Culex*, and *Anopheles*, which facilitate spread between infected animals and humans [172]. Humans are typically infected through direct or indirect contact with blood, tissues, or body fluids of infected animals, particularly during slaughtering, butchering, veterinary procedures, or handling of aborted animal materials. Vector-borne transmission plays a central role during epizootic amplification phases. Human-to-human transmission is extremely rare and has only been reported in limited settings, mainly laboratory or healthcare-associated exposures, including aerosol or contaminated material contact under specific conditions [167].

#### 4.4.3. Clinical Features

In humans, RVF infections are usually asymptomatic or manifest as mild influenza-like illness with fever, headache, myalgia, and malaise. Severe complications, though rare, include hemorrhagic fever, encephalitis, and ocular disease, which can lead to long-term visual impairment [172]. In animals, particularly sheep, goats, and cattle, the infection can cause high mortality, neonatal deaths, and abortions, leading to substantial socio-economic impact. Human immune responses involve innate mechanisms, such as interferon signaling and activation of dendritic cells, as well as adaptive immunity with neutralizing antibodies targeting glycoproteins Gn and Gc. CD8+ T-cell responses are important for viral clearance in animal models [169,170].

#### 4.4.4. Vaccination and Treatment

No specific antiviral treatment exists for RVF, so prevention is critical. Vaccines have been developed for animals, including live-attenuated (*Smithburn strain*) and inactivated formulations, which reduce viral circulation in livestock and indirectly protect humans. For humans, vaccine development is ongoing, with candidates including live-attenuated, inactivated, and viral-vectored platforms evaluated in preclinical and clinical studies. Protective immunity is associated with the production of neutralizing antibodies against Gn/Gc glycoproteins [170,171]. Additional preventive measures include vector control, use of personal protective equipment when handling animals, and avoiding contact with blood or tissues of infected livestock. Monitoring of climatic conditions and host populations is also essential for predicting and mitigating outbreaks.

#### 4.4.5. Future Challenges

Despite its relatively low capacity for sustained human-to-human transmission, RVF remains a significant zoonotic threat due to its epidemic potential in animal populations and strong dependence on ecological conditions. Climate change, extreme rainfall events, and changes in land use may expand vector habitats and increase outbreak frequency. Future research priorities include improved predictive modelling of outbreaks, development of safe and effective human vaccines, and strengthened One Health-based surveillance integrating veterinary, environmental, and human health data. Enhanced vector monitoring and early warning systems are essential for mitigating the impact of future RVF epidemics on both public health and food security.

## 5. Strategies for Preventing Emerging Viral Outbreaks: Surveillance, Immunization, and Education

### 5.1. One Health Framework: Interconnectedness and Policy Strategies for Zoonotic Disease Surveillance

**Figure 4:**
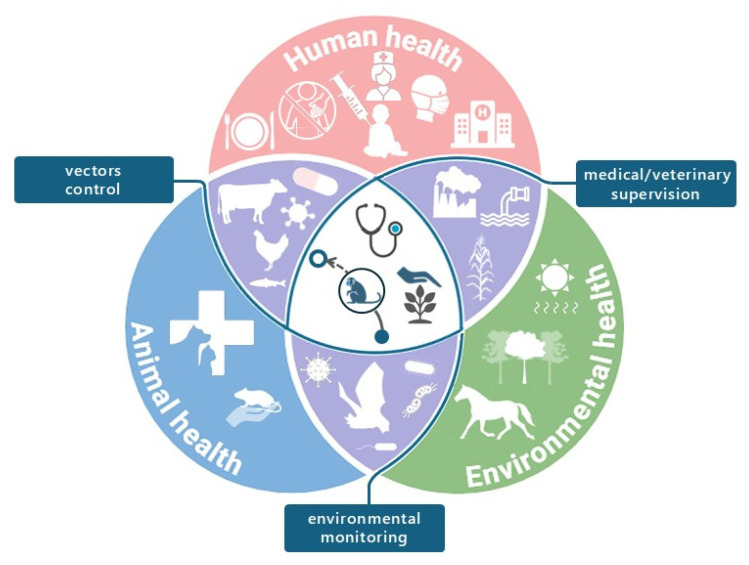
One Health framework for zoonotic disease control. The three primary pillars—human health, animal health, and environmental health—represent complementary domains where targeted interventions can mitigate disease emergence and spread. Human health measures include epidemiological surveillance, vaccination programs, and clinical management of cases. Animal health focuses on monitoring wildlife and domestic reservoirs, along with veterinary surveillance to detect and contain infections. Environmental health encompasses ecosystem monitoring and assessment of climate and land-use changes that influence pathogen dynamics. At the center of the framework, integrated surveillance and outbreak response highlight the synergy achieved when data from all three domains are combined, enabling timely detection, risk assessment, and coordinated intervention to prevent zoonotic spillover and limit outbreak impact. The figure was created in part using BioRender.com.

The One Health framework is a holistic, multidisciplinary approach that recognizes the intricate connections between human health, animal health, and environmental systems. It is grounded in the understanding that the majority of emerging infectious diseases, particularly viral zoonoses, originate at the interface of these domains [172,173,174]. By integrating knowledge across medical, veterinary, and ecological disciplines, One Health provides a comprehensive perspective on disease emergence, spillover dynamics, and transmission pathways, facilitating a more complete understanding of how pathogens traverse species barriers. Zoonotic viruses frequently emerge as a result of interactions among wildlife reservoirs, domestic animals, and human populations, often driven by environmental changes such as deforestation, urbanization, agricultural expansion, and climate change [17,175,176]. These complex interactions underscore the necessity for a multidisciplinary perspective in infectious disease research and control, where surveillance and intervention efforts are coordinated across sectors and ecosystems (Figure 4).

One Health enhances early detection of spillover events, guides risk assessment, and informs the design of targeted interventions to prevent further transmission [174,177]. Operationalizing One Health from a policy standpoint requires strong cross-sectoral collaboration, harmonized data collection, and shared surveillance infrastructure that encompasses wildlife, livestock, and human populations as well as environmental determinants of pathogen persistence and spread [172,173]. Policies should promote information exchange between veterinary and human health authorities, and coordination across local, national, and international stakeholders, to enable rapid responses to emerging threats [178]. In addition to surveillance, the One Health approach emphasizes preventive strategies such as coordinated vaccination programs, public education on safe human–animal interactions, and ecological management to reduce pathogen exposure and transmission risk [179,180,181]. By bridging scientific research and policy planning, One Health strengthens outbreak preparedness, informs resource allocation, and supports sustainable interventions that address the root causes of zoonotic emergence. Ultimately, this framework provides a strategic foundation for mitigating the impact of emerging zoonotic diseases and improving global health security in an interconnected world.

### 5.2. Integrating Digital Technologies and Genomics for Viral Outbreak Detection

The rapid emergence and global spread of infectious diseases, particularly viral zoonoses, have underscored the imperative for innovative, data-driven approaches to epidemic surveillance and outbreak response. Digital epidemiology and genomic surveillance have emerged as complementary methodologies that enhance early detection, continuous monitoring, and timely intervention, thereby improving preparedness for both local and global health threats [182,183,184]. Digital epidemiology leverages non-traditional data streams, including electronic health records, participatory reporting platforms, mobile applications, syndromic surveillance systems, and social media trends. These sources capture real-time or near-real-time signals of unusual disease activity that traditional surveillance may miss or detect only after delays [185,186]. Artificial intelligence (AI) and machine learning (ML) algorithms can process and analyze these large, heterogeneous datasets to predict outbreak hotspots, assess changing transmission dynamics, and support resource prioritization for public health interventions [187,188,189]. During the COVID-19 pandemic, AI-driven analyses of mobility data, symptom reporting, and online search trends provided crucial insights into emerging clusters and transmission patterns ahead of conventional case reporting [190,191]. Similarly, in the 2022 mpox outbreak, digital reporting platforms enabled rapid case identification and trend analysis across non-endemic regions, helping authorities to target vaccination, contact tracing, and community outreach measures [192,193].

Genomic surveillance provides high-resolution insights into pathogen evolution, diversity, and transmission chains. Whole-genome sequencing (WGS) allows precise identification of viral mutations, tracking of emerging variants, and reconstruction of transmission linkages in near real time [194,195]. For SARS-CoV-2, genomic surveillance was pivotal in detecting variants of concern such as Alpha, Delta, and Omicron and informing vaccine update strategies [196,197]. In Ebola outbreaks, sequencing data helped trace transmission chains and differentiate local persistence from new introductions, thus optimizing containment efforts [198,199]. For mpox, genomic analyses have clarified lineage relationships, tracked multiple introductions into non-endemic regions, and informed tailored public health strategies to mitigate spread [200,201].

The integration of digital and genomic approaches allows proactive outbreak detection and response. Combining AI-driven analytics with sequencing enhances the ability to identify emerging pathogens, monitor variant spread, and predict potential spillover from animal reservoirs. Real-time case reporting aligned with genomic monitoring supports targeted vaccination campaigns, precise contact tracing, and refined risk assessment—especially crucial when dealing with viruses capable of rapid adaptation or cross-species transmission [195,202].

Beyond conventional vaccination strategies aimed at protecting susceptible hosts, increasing attention has been directed toward preventive approaches that target pathogen transmission itself [203,204]. Transmission-blocking vaccines, which interfere with pathogen development, survival, or transmission within arthropod vectors, are being explored as complementary tools for controlling vector-borne diseases. In parallel, growing evidence suggests that manipulation of vector-associated microbiota may alter vector competence by reducing pathogen colonization and transmission efficiency [205,206]. Although these approaches remain largely at the experimental stage, they highlight promising new avenues for interrupting transmission cycles and may complement existing vaccination, surveillance, and vector-control strategies. Continued advances in vector biology, microbiome research, and immunology are expected to support the development of innovative transmission-focused interventions within the broader One Health framework.

From a policy and public health perspective, operationalizing digital epidemiology and genomic surveillance requires robust infrastructure, cross-sectoral collaboration, and interoperable data-sharing frameworks. Harmonizing data standards across human, animal, and environmental health sectors aligns with the One Health approach, ensuring coordinated detection and response across disciplines [207,208]. Regulatory frameworks should enable rapid information exchange, support joint response mechanisms, and facilitate international collaboration, as zoonotic threats often transcend borders. Preventive strategies under this integrated framework include vaccination programs tailored to emerging variants, public education on human–animal interactions, ecological management to reduce pathogen exposure, and resource allocation based on dynamic risk profiles. By bridging scientific evidence and policy planning, digital epidemiology and genomic surveillance enhance outbreak preparedness, support sustainable interventions, and reduce the global impact of emerging zoonotic viruses. Ultimately, leveraging AI, big data, and genomic technologies within a One Health context strengthens global capacity to detect, prevent, and respond to infectious disease emergence, enhancing public health security in an increasingly interconnected world [24].

### 5.3. Community Engagement and Risk Communication in Zoonotic Outbreaks

Effective public communication and community engagement are essential components of epidemic preparedness and response, particularly for zoonotic viral outbreaks such as monkeypox, SARS-CoV-2, and Ebola. Misinformation and distrust in public health guidance can undermine containment efforts, reduce vaccine uptake, and hinder the implementation of preventive measures [209,210]. Building trust requires transparent, culturally sensitive communication strategies that address community concerns and encourage adherence to recommended interventions [211].

Fighting misinformation is a critical challenge. Social media, informal networks, and rapid online dissemination of unverified information can amplify misconceptions about disease severity, transmission, or vaccination safety [212,213]. Public health authorities must proactively monitor misinformation trends, respond with evidence-based messaging, and collaborate with trusted community leaders to ensure accurate information reaches target populations. Educational campaigns should be tailored to local contexts, using accessible language and culturally relevant examples to enhance comprehension and engagement [214]. Building trust in vaccination and preventive measures is equally crucial. Past experiences with vaccination campaigns highlight the importance of engaging communities early, addressing fears, and providing transparent information about vaccine safety and efficacy [215,216]. In outbreak settings, trust fosters community cooperation with contact tracing, quarantine, and prophylactic interventions, ultimately improving public health outcomes.

International agencies, including the World Health Organization (WHO), the U.S. Centers for Disease Control and Prevention (CDC), Africa CDC, and global consortia, play a key role in guiding communication strategies, coordinating responses, and providing evidence-based recommendations. They also support countries with limited infrastructure, ensuring that messaging and interventions are consistent, credible, and culturally appropriate. Addressing vaccine and resource inequality is a fundamental aspect of community engagement. Equitable distribution of vaccines, diagnostics, and therapeutics reduces disparities in outbreak impact and enhances global health security [217]. Strategies include prioritizing high-risk populations, supporting local healthcare capacity, and leveraging international partnerships to ensure timely access to life-saving interventions. Effective public communication and community engagement are indispensable for controlling emerging zoonotic diseases. By combating misinformation, fostering trust, and addressing inequalities, public health authorities can promote compliance with preventive measures, optimize vaccination coverage, and strengthen resilience against future outbreaks. Integrating these strategies with surveillance, genomic monitoring, and the One Health framework enhances the overall effectiveness of epidemic response efforts.

### 5.4. Global Vaccine Equity: Policy Approaches and Implementation Strategies

Equitable access to vaccines is a critical component of global health security and pandemic preparedness. Vaccination programs are estimated to prevent approximately 4–5 million deaths annually worldwide and remain one of the most cost-effective public health interventions [218,219]. Despite these benefits, substantial disparities in vaccine availability and coverage persist between high-income countries and low- and middle-income countries (LMICs). These inequalities were particularly evident during the COVID-19 pandemic, when high-income countries secured the majority of early vaccine supplies through advance purchase agreements, leaving many LMICs with limited or delayed access [220].

Global vaccine inequity arises from a complex interplay of structural, economic, and logistical factors (Table 7). Vaccine supply is highly concentrated among a small number of manufacturers located primarily in high-income regions, which creates dependence on global supply chains and contributes to unequal distribution during health emergencies [221]. During the early phases of the COVID-19 vaccine rollout, more than half of all available doses were reserved by a small group of wealthy nations representing a minority of the global population [220]. Such disparities can prolong outbreaks, increase the risk of pathogen evolution, and ultimately undermine global disease control efforts [222].

**Figure 5:**
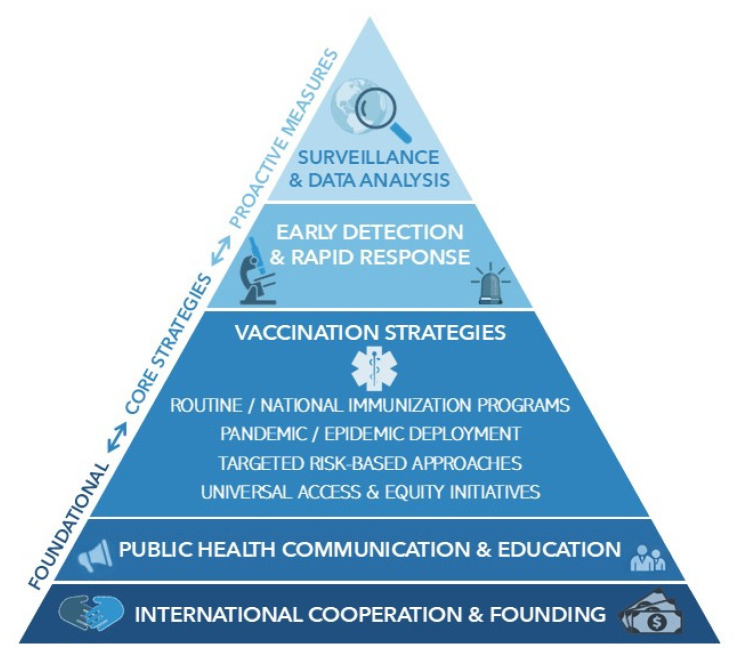
Layered strategies for emerging zoonotic disease preparedness. Surveillance and early detection enable rapid identification of outbreaks. Vaccination strategies include targeted immunization of high-risk groups, ring vaccination, and adaptable vaccine platforms. Public communication fosters trust and compliance, while international cooperation ensures coordinated response and equitable vaccine distribution.

Infrastructure limitations further exacerbate vaccination challenges in resource-constrained settings. Effective vaccine deployment requires reliable cold-chain systems, adequate transportation networks, trained healthcare personnel, and robust immunization registries. Many LMICs face shortages in these areas, particularly in rural and remote regions where healthcare access is limited [230,231]. Weak health systems may also hinder the ability to conduct large-scale immunization campaigns, maintain vaccine storage standards, and track vaccination coverage. Socioeconomic and sociocultural barriers also influence vaccine uptake. Individuals from lower socioeconomic backgrounds often experience reduced access to vaccination services and may face additional barriers such as transportation limitations, lower health literacy, and reduced trust in healthcare systems [227,232]. Vaccine hesitancy, driven by misinformation, historical distrust, or lack of access to reliable information, can further reduce vaccination coverage in vulnerable populations. Community engagement, transparent communication, and culturally tailored education are essential to increase vaccine confidence and uptake [228] (Figure 5).

To address these challenges, several global mechanisms have been established to promote equitable vaccine distribution. The COVAX Facility, co-led by the World Health Organization (WHO), Gavi, and the Coalition for Epidemic Preparedness Innovations (CEPI), was designed to ensure fair access to vaccines during the COVID-19 pandemic. By pooling procurement and financing, COVAX aimed to distribute vaccines equitably among participating countries, particularly those with limited purchasing power [220]. Although the initiative has delivered hundreds of millions of doses worldwide, it has faced challenges related to supply shortages, funding gaps, and export restrictions [233,234].

Expanding regional vaccine manufacturing capacity has been identified as a key strategy for improving vaccine equity. Strengthening local production capabilities in Africa, Latin America, and Asia can reduce dependency on global supply chains and improve access during public health emergencies [223]. Technology transfer initiatives, intellectual property sharing mechanisms, and collaborative manufacturing agreements are increasingly recognized as critical tools to facilitate this transition. In addition to manufacturing capacity, strengthening health system infrastructure is essential for improving vaccine delivery. Investments in cold-chain systems, workforce training, digital health technologies, and mobile immunization units can significantly enhance vaccination coverage and program efficiency [235,236]. Digital immunization registries and integrated surveillance systems support real-time monitoring of vaccine distribution and uptake, enabling targeted public health interventions [237].

Ethical allocation frameworks also play an important role in guiding equitable vaccine distribution. Public health strategies increasingly emphasize prioritizing high-risk populations—including healthcare workers, elderly individuals, and people with underlying medical conditions—as well as marginalized communities that may experience disproportionate disease burden [222]. Such strategies not only reduce mortality but also contribute to more equitable health outcomes.

International cooperation remains essential for addressing vaccine inequality and strengthening global pandemic preparedness. Multilateral partnerships involving governments, international organizations, global health initiatives, and regional public health agencies—facilitate resource sharing, coordinated policy development, and technical assistance for vaccination programs. Strengthening these collaborative frameworks is crucial to ensuring that future vaccine development and distribution efforts are more equitable and responsive to global needs [238].

## 6. Conclusions

Emerging viral zoonoses continue to pose a significant and evolving challenge to global public health. The example of monkeypox illustrates how complex interactions among animal reservoirs, environmental changes, human behaviors, and viral evolution can drive pathogen spillover and facilitate international spread. These dynamics underscore the critical importance of integrated surveillance systems, early warning platforms, and coordinated public health responses within a One Health framework.

Vaccination remains a cornerstone strategy for controlling zoonotic outbreaks and reducing disease burden. However, future vaccine development should move toward more innovative and adaptable platforms, including broadly protective, next-generation, and rapidly deployable technologies capable of responding to emerging and re-emerging pathogens. In parallel, strengthening genomic surveillance and integrating digital epidemiology approaches will be essential for real-time outbreak detection, monitoring viral evolution, and guiding timely interventions. Future research should also prioritize the development of novel preventive strategies aimed not only at protecting hosts but also at interrupting pathogen transmission. These include transmission-blocking approaches, vector-targeted interventions, and emerging microbiota-based strategies targeting vector competence. Expanding knowledge of host–pathogen–vector interactions, including vector immunity and microbiota composition, may further support the design of innovative control measures. Effective preparedness will require multidisciplinary and globally coordinated efforts integrating vaccination programs, surveillance systems, environmental monitoring, and strategic public communication. Implementation of the One Health approach, linking human, animal, and environmental health, is essential for strengthening outbreak prevention and response. Ultimately, global collaboration, resilient health systems, and equitable access to vaccines and preventive tools will be crucial to reducing the impact of emerging viral zoonoses and enhancing preparedness for future pandemics.

## Figures and Tables

**Table 1:** Clinical differentiation of monkeypox from other viral infections based on [92,96,97,98,99].

Clinical Feature	Monkeypox	Chickenpox	Smallpox	Shingles	Cold Sore
Etiological factor	Monkeypox virus	Varicella-zoster virus	Variola virus	Varicella-zoster virus	Herpes simplex virus
Incubation period	7–14 days	10–21 days	7–17 days	7–21 days	2–12 days
Prodromal symptoms	fever, muscle pain, lymphadenopathy	fever, weakness	sudden fever, pain, rash	pain in the dermatome, burning sensation	burning sensation, itching
Location of lesions	face, limbs, hands, feet, genitals, mucous membranes	face, torso, scalp	face, torso, limbs	usually torso or head (unilateral)	lips, genitals, fingers areas
Number of lesions	from a dozen to a several dozen	usually very severe	numerous and densely arranged	usually limited	usually limited, often grouped locally
Type of lesions	spots, papules, vesicles, pustules, scabs	spots, papules, vesicles, scabs	papules, blisters, pustules	blisters, pustules, sometimes hemorrhagic	blisters on reddened skin
Stages of lesions development	synchronized	non-synchronized	synchronized	synchronized (locally)	synchronized
Mucosal lesions	frequent (oral cavity, genitals)	often	rare	sometimes (oral cavity)	very often (oral cavity, genitals)
Lymphadenopathy	present	none or mild	usually absent	usually absent	only with primary infection
Painfulness of lesions	often painful (especially genitals)	minor	possible	often severe pain, neuralgia	burning, pain, itching

**Table 2:** Comparison of MVA-BN and LC16m8 vaccines for monkeypox prevention based on [104,105,106].

Feature	MVA-BN Vaccine	LC16m8 Vaccine
Virus	live, unable to replicate	live, weakly replicating
Multiplication in human cells	no	yes (limited)
Safety	very high	very good, but lower than MVA-BN
Approval for mpox	yes	not officially registered
WHO status	first-choice vaccine	recommended as an alternative in the absence of MVA-BN

**Table 3:** Summary of major avian influenza A virus (AIV) subtypes of zoonotic concern (based on [115,116,117,118,119,120,121]).

Subtype	Hosts & Ecology	Human Infection Features	Reassortment & Evolution	Pandemic Risk
H5N1	Wild birds, poultry; widespread globally across Asia, Europe, Africa, Americas	Sporadic human cases via direct/indirect contact; high case fatality; no sustained human-to-human spread reported	Segmented genome enables reassortment with other AIVs; evolving genotypes detected worldwide	Humans lack pre-existing immunity; pandemic potential if sustained transmission evolves; surveillance critical
H7N9	Circulated in poultry in China; first detected in 2013	Human infections linked to live poultry exposure; severe respiratory disease; no consistent human-to-human transmission	Frequent reassortment with H9N2 internal genes, contributing to human epidemic waves	High virulence in humans; continuing evolution necessitates monitoring
H9N2	Endemic in poultry worldwide	Occasional human infections; typically mild or asymptomatic, linked to poultry exposure (reports continue, including recent cases)	Acts as a gene donor through reassortment to other subtypes, enhancing zoonotic potential of viruses like H7N9	Low direct human pathogenicity but important contributor to reassortant emergence
Other AIVs (e.g., H5N6, H10N3, H3N8)	Detected in birds and occasionally mammals	Sporadic human cases reported (e.g., H5N6, H10N3)	Some reassort with other AIVs; genetic diversity arises through coinfection	Continue to pose sporadic zoonotic risk; require ongoing surveillance (WHO reports)

**Table 4:** Comparison of avian influenza vaccine platforms and strategies.

Vaccine Platform/Strategy	Mechanism	Immunogenicity/ Protective Effect	Advantages	Limitations	References
Inactivated AIV vaccines (e.g., H7N9 IIV)	Killed virus induces antibody response	Induces strain-specific antibodies; phase II trials have evaluated dosing and adjuvant effects	Established technology; relatively safe	Variable immunogenicity, may require adjuvants and booster doses	[133,134,135]
mRNA vaccines (H5N1)	Encodes HA (±NA) to stimulate humoral and cellular immunity	Robust neutralizing antibodies, protection in ferrets and reduced transmission; strong immunogenicity in preclinical models	Rapid development; scalable; elicits both humoral and cellular responses	Not yet licensed for AIV in humans; clinical data limited	[136,137]
mRNA vaccines (avian strains in poultry)	Encodes HA to protect birds	Complete protection against homologous challenges in chickens, moderate heterologous protection	Useful for animal health and spillover reduction	Efficacy may vary across strains	[138]
DNA/HLAII-targeted vaccine (H7N9)	DNA vaccine targets antigen to HLAII to stimulate adaptive immunity	Protective in mice and ferrets in challenge models	Strong T-cell responses; alternative platform	Early experimental stage; human data lacking	[139]
Prime-boost regimens	Combinations of platforms (e.g., inactivated + adjuvant or DNA + protein)	Can enhance breadth and durability of immune responses	Potentially stronger protection across subtypes	Complex schedules; not widely implemented	[140,141]
Public health strategy: occupational vaccination	Targeted vaccination of high-risk groups	Reduces exposure risk where outbreaks occur	Protects workers in close contact with infected birds	Not general population coverage; relies on risk assessment	[142,143]

**Table 5:** Summary of key features, transmission, and prevention of Ebola and Marburg virus diseases based on [144,145,150,151,152,153,154].

Feature	Ebola Virus Disease (EVD)	Marburg Virus Disease (MVD)
Etiological agent	*Ebolavirus*, mainly *Zaire ebolavirus* (EBOV)	*Marburgvirus*, *Marburg virus* (MARV)
Reservoir	Fruit bats (Pteropodidae family), possibly other wildlife	Egyptian rousette bats (Rousettus aegyptiacus), possibly other bats
Primary hosts/ecology	Spillover via direct contact with infected animals; human outbreaks primarily in Africa	Spillover via caves/mines inhabited by bats; human outbreaks primarily in Africa
Transmission to humans	Direct contact with blood, tissues, or body fluids of infected animals; handling of bushmeat	Direct contact with infected bats, tissues, or body fluids; contaminated surfaces
Human-to-human transmission	Contact with blood, body fluids (saliva, urine, sweat, vomit, semen, breast milk), contaminated objects	Contact with blood, body fluids, or contaminated objects; no sustained droplet transmission
Incubation period	2–21 days (commonly 8–10 days)	2–21 days (commonly 5–10 days)
Clinical features	Fever, headache, myalgia, fatigue, gastrointestinal symptoms, rash, hemorrhagic manifestations, multiorgan failure	Fever, headache, myalgia, fatigue, gastrointestinal symptoms, hemorrhage, multiorgan failure
Case fatality rate	25–90% depending on outbreak and strain	~50–88% depending on outbreak
Treatment	Supportive care (fluids, hemodynamic support, oxygen, treat secondary infections); investigational antivirals (remdesivir, monoclonal antibodies)	Supportive care only; investigational antivirals under study
Prophylaxis	Licensed vaccines: rVSV-ZEBOV (Ervebo), Zabdeno/Mvabea; used in outbreak response and high-risk populations	No licensed vaccine currently; investigational vaccines in development
Pandemic potential	High lethality, potential for local outbreaks; controlled with rapid response, vaccination, infection control	High lethality; low frequency outbreaks; monitoring and rapid containment essential

**Table 6:** Rift Valley Fever (RVF) characteristics and zoonotic risk based on [165,166,167,168,169,170,171].

Feature	Rift Valley Fever (RVF)
Etiological agent	*Phlebovirus*, family *Phenuiviridae*
Reservoir	Wild ruminants, possibly other wildlife; maintained in mosquitoes (*Aedes*, *Culex*, *Anopheles*)
Primary hosts/ecology	Domestic and wild ruminants act as amplifying hosts; outbreaks associated with heavy rainfall and mosquito population surges
Transmission to humans	Direct contact with blood, tissues, or body fluids of infected animals; exposure during slaughter, veterinary care, or handling infected carcasses; mosquito-borne transmission possible
Human-to-human transmission	Very rare; primarily limited to laboratory or healthcare settings (aerosol or contaminated surfaces)
Incubation period	2–6 days (commonly 3–5 days)
Clinical features	Mild influenza-like illness in most cases; severe complications include hemorrhagic fever, encephalitis, ocular disease, and rare fatal outcomes
Case fatality rate	<1% in mild cases; up to 10–20% in severe complications, depending on outbreak and population
Treatment	Supportive care only; no specific antivirals licensed for humans
Vaccination/prophylaxis	Licensed veterinary vaccines (live-attenuated Smithburn, inactivated vaccines) reduce animal infection; human vaccines under development; prevention relies on vector control, PPE, and safe handling of animals
Pandemic potential	Low human-to-human transmission limits pandemic potential; high impact on livestock and food security; climate change, flooding, and host density influence outbreak risk

**Table 7:** Key Barriers and Strategies for Equitable Vaccine Access.

Feature	Barriers	Strategies	References
Vaccine supply	Concentration of production in high-income countries; dependence on global supply chains; limited doses in LMICs	Expand regional manufacturing capacity; technology transfer; collaborative production agreements	[220,223,224]
Infrastructure	Limited cold-chain storage; insufficient transportation networks; lack of trained personnel; weak health systems	Investment in cold-chain systems, transportation, workforce training; mobile vaccination units; integrated surveillance and digital registries	[221,225]
Socioeconomic and cultural barriers	Low health literacy, transportation limitations, reduced access in rural/remote areas; vaccine hesitancy	Community engagement; culturally tailored education campaigns; targeted outreach to vulnerable populations	[226,227,228]
Vaccine hesitancy	Distrust in authorities; misinformation on social media; historical distrust	Transparent communication; trusted local leaders; evidence-based messaging; participatory approaches	[222,228]
Global coordination	Fragmented policy approaches; inequitable procurement; export restrictions	Initiatives like COVAX; multilateral partnerships (WHO, Gavi, CEPI, UNICEF); harmonized global policies	[220,224]
Ethical allocation	Risk of marginalized groups being overlooked; prioritization challenges	Ethical frameworks prioritizing high-risk and vulnerable populations; equity-focused vaccination policies	[222,229]
Pandemic preparedness	Slow response in LMICs; limited surge capacity	Strengthen surveillance and immunization systems; advance planning for rapid deployment; integration with One Health approaches	[221,225]

## Data Availability

The data presented in this study are openly available under reference numbers.

## Abbreviations

The following abbreviations are used in this manuscript:

rVSV-ZEBOV	recombinant Vesicular Stomatitis Virus-Zaire Ebola Virus
SARS-CoV-2	Severe Acute Respiratory Syndrome Coronavirus 2
MERS-CoV	Middle East Respiratory Syndrome Coronavirus
MVA-BN	Modified Vaccinia Ankara-Bavarian Nordic
GISRS	Global Influenza Surveillance and Response System
LMICs	Low- and Middle- Income Countries
EIDs	Emerging Infectious Diseases
STEC	Shiga Toxigenic *E. coli*
MPXV	Monkeypox virus
MARV	*Marburg* virus
EBOV	*Zaire ebolavirus*
RVFV	Rift Valley Fever Virus
Mpox	Monkeypox
MSM	Men who have sex with men
EMA	European Medicines Agency
AIV	Avian Influenza Virus
WHO	World Health Organization
EVD	Ebola Virus Disease
MVD	Marburg Virus Disease
RVF	Rift Valley Fever
WGS	Whole-Genome Sequencing
CDC	Centers for Disease Control and Prevention
HA	Hemagglutinin
NA	Neuraminidase
NK	Natural Killer
AI	Artificial Intelligence
ML	Machine Learning
